# Out of the stable: Social disruption and concurrent shifts in the feral mare (*Equus caballus*) fecal microbiota

**DOI:** 10.1002/ece3.10079

**Published:** 2023-05-11

**Authors:** Grace J. Vaziri, Maggie M. Jones, Haley A. Carr, Cassandra M. V. Nuñez

**Affiliations:** ^1^ Ecology and Evolutionary Biology University of Connecticut Mansfield Connecticut USA; ^2^ Department of Natural Resource Ecology and Management Iowa State University Ames, Iowa USA; ^3^ Department of Biological Sciences The University of Memphis Memphis Tennessee USA; ^4^ Present address: School of Natural Resources and Environment University of Florida Gainesville Florida USA

**Keywords:** alpha diversity, beta diversity, feral horse, microbiome, microbiota, social disruption

## Abstract

The disruption of animals' symbiotic bacterial communities (their microbiota) has been associated with myriad factors including changes to the diet, hormone levels, and various stressors. The maintenance of healthy bacterial communities may be especially challenging for social species as their microbiotas are also affected by group membership, social relationships, microbial transfer between individuals, and social stressors such as increased competition and rank maintenance. We investigated the effects of increased social instability, as determined by the number of group changes made by females, on the microbiota in free‐living, feral horses (*Equus caballus*) on Shackleford Banks, a barrier island off the North Carolina coast. Females leaving their groups to join new ones had fecal microbial communities that were similarly diverse but compositionally different than those of females that did not change groups. Changing groups was also associated with the increased abundance of a several bacterial genera and families. These changes may be significant as horses are heavily dependent upon their microbial communities for nutrient absorption. Though we cannot identify the particular mechanism(s) driving these changes, to the best of our knowledge, ours is the first study to demonstrate an association between acute social perturbations and the microbiota in a free‐ranging mammal.

## Graphical abstract

We investigated the effects of increased social instability, as determined by the number of group changes made by females, on the microbiota in free‐living, feral horses (*Equus caballus*) on Shackleford Banks, a barrier island off the North Carolina coast. Females leaving their groups to join new ones had fecal microbial communities that were compositionally different than those of females that did not change groups.

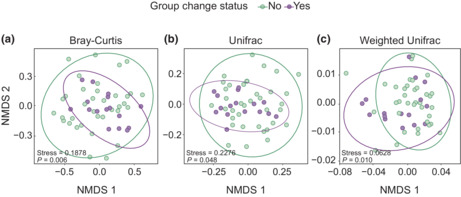

## 1 INTRODUCTION

Links between the disruption of animals' microbial communities (their symbiotic bacterial communities) and variations in diet, hormone levels, and environmental factors are ubiquitous among both solitary and social species (Comizzoli et al., 2021; Zaneveld et al., 2017). However, group‐living species must also contend with additional, uniquely social factors that could influence their microbiota, including changes to group composition, their relationships within the group, and the microbial transfer between various individuals. In adult yellow baboons (*Papio cynocephalus*), interaction rates explained variation in the gut microbiome (even after controlling for diet, kinship, and shared environments), strongly implicating direct physical contact among social partners in the transmission of gut microbial species (Tung et al., 2015). Similarly, in semi‐feral Welsh ponies (*Equus ferus caballus*), microbiota communities varied with spatial structuring and even with specific social interactions within groups (including those between males and females and mothers and offspring; Antwis et al., 2018).

In addition, various stressors including illness, environmental stressors, and physical trauma (experienced by virtually all species at one time or another) have been linked to microbial disruption in several taxa (Zaneveld et al., 2017). Factors as disparate as foraging in degraded habitats (howler monkeys, *Alouatta pigra*), pathogen infection (bullfrogs, *Lithobates catesbeianus*), and increases in temperature (corals, *Anthozoa* spp.) can all affect species' microbiota (Amato et al., 2013; Zaneveld et al., 2016). Social species experience similar stressors, in addition to those unique to group‐living, including increased competition (for both food/water resources and mates), the maintenance of one's rank within and/or between groups, and group “politics” (Ward & Webster, 2016), all of which can potentially affect their microbiotas. Links between social stressors and the microbiota have been well‐established in the laboratory. Mice (*Mus musculus* spp.) subject to prolonged aggression and infant rhesus monkeys (*Macaca mulatta*) subject to maternal separation show alterations to their microbiota (Bailey et al., 2010; Bailey & Coe, 1999). In the barn, domestic horses (*Equus* spp.) subject to transport or weaning and those with undifferentiated colitis (inflammation of the caecum and colon) all show changes to their microbiota (Costa et al., 2012; Mach et al., 2017; Schoster et al., 2016). Such alterations, often including decreased microbial diversity and stability, may reduce animals' ability to fight off infection (Bailey, 2012), leaving them more susceptible to a host of enteric pathogens (Bailey et al., 2010, Bailey & Coe, 1999). Similar links have been established in the wild. Both red squirrels (*Tamiasciurus hudsonicus*) experiencing trapping stress and yellow‐legged gulls (*Larus michahellis*) with experimentally elevated corticosterone levels show decreased microbiota diversity. Corals show myriad changes with simulated overfishing, nutrient pollution, and increased temperature (Noguera et al., 2018; Stothart et al., 2016; Zaneveld et al., 2016). Similarly, howler monkeys living in degraded habitats exhibit less diverse microbial communities; though these changes were attributed largely to dietary differences, the authors concede that increases in stress hormones might have played an important role (Amato et al., 2013).

Here, we investigate whether increased social instability is associated with changes in the microbiota in a population of free‐living, feral horses (*Equus caballus*) living on Shackelford Banks, North Carolina, USA. Feral horses organize themselves into groups (bands) which contain the male(s)—or stallion(s), his female(s)—or mare(s), and their offspring (Feist & McCullough, 1976; Linklater et al., 2000; Rubenstein, 1981, 1986). These groups are normally long‐lasting with few changes to group composition save for the dispersal of offspring (Klingel, 1975). Mares will leave their males/groups to join new ones primarily in response to a band stallion's attributes, including age, rank, length of tenure, and to a lesser extent, his propensity to engage in male–male aggression (Rubenstein, 1994; Rutberg, 1990), but at a fairly low rate (0%–17% of females; Berger, 1977, Feist & McCullough, 1976, Linklater et al., 2000, Rubenstein, 1981). This stability of group membership is important to the overall well‐being of all group members; decreases in stability have been associated with decreased body condition and reproductive success, and increased parasite load and offspring mortality (Kaseda et al., 1995; Linklater et al., 1999) all of which could affect animals' microbiotas (Comizzoli et al., 2021; Zaneveld et al., 2017). Moreover, the individual changing groups will experience a new home range and potentially changes in forage and water access, exposure to the microbial communities of their new bandmates, and a reassessment/reestablishment of their rank; again, all of which can affect their microbiota (Antwis et al., 2018; Zaneveld et al., 2017).

Decreases to the social stability typical of feral horses have been associated with immunocontraceptive treatment (Madosky et al., 2010; Nuñez et al., 2009, 2017; Ransom et al., 2010). From 2000 to 2009, the feral horse population of Shackelford Banks, North Carolina was managed with the immunocontraceptive vaccine, porcine zona pellucida (PZP). Briefly, the vaccine induces an immune response which stimulates the production of antibodies that bind sperm receptors on the egg's surface, thereby blocking fertilization while still allowing ovulation and the associated cycles of sex hormones (Sacco, 1977). Typically, anti‐PZP titers only remain above control levels for approximately 10 months (Willis, 1994), though one PZP treatment can effect subfertility for up to three breeding seasons (Turner Jr. et al., 2007). Mares treated with PZP can be 10 times as likely to switch bands, leaving their male to join another (Nuñez et al., 2009), thereby decreasing band stability and effecting some level of social disruption. At the time of this study (6–7 years after the contraception program was largely suspended), Shackelford mares that previously received PZP continued to change groups more often compared to mares that had never been treated (Jones & Nuñez, 2019; Nuñez et al., 2017). Evidence suggests that these prolonged effects are due to continued subfertility and not any lingering effect of the treatment itself (Nuñez et al., 2017). This increased variability in group changing behavior afforded us the unique opportunity to better discern the potential links between social disruption and microbiota in the wild.

In social species, significant changes in the behavior of individuals or subgroups of individuals often affects that of their close associates (Ward & Webster, 2016). Stallions respond to female group changing behavior aggressively with chases, and sometimes kicks and bites to the females as they attempt to bring them back to the group (Madosky, 2011). Moreover, females engaging in this behavior often find themselves near highly escalated male–male conflicts (Jones & Nuñez, 2019). In addition, females that manage to change groups are often subject to aggression from resident females (C. M. V. Nuñez, unpublished data). Perhaps unsurprisingly, group changing behavior is associated with an endocrine stress response (Nuñez et al., 2014) which is reliably assessed via fecal cortisol metabolite (FCM) concentrations (Mostl & Palme, 2002; Nuñez et al., 2014; Wasser et al., 2000). Mares engaging in group switching exhibited higher FCM concentrations than those that did not. Moreover, mares engaging in the behavior more often exhibited higher FCM concentrations than those doing so less often. In some cases, FCM concentrations remained elevated for at least 2 weeks post‐behavior (on average) indicating at least some lasting effect of the behavior (and/or its associated stressors) on mare stress physiology.

We sought to determine whether group switching behavior, that is, increased social instability, was correlated with changes in the mare's gut microbiota. Using fecal samples gathered in the field (2015 and 2016), we examined the microbiota communities of mares that did and did not change groups to determine links between a relevant social behavior (and potential stressor) and the diversity and composition of the microbiota. We predicted that mares changing groups would exhibit decreased diversity to their microbiota communities when compared to mares that did not change groups (Bailey et al., 2011). We also expected that mares changing groups would have compositionally different (shifted) fecal bacterial communities and would host bacterial communities that were more variable than those that did not (Zaneveld et al., 2017).

## 2 MATERIALS AND METHODS

### 2.1 Study area

We conducted this study on Shackleford Banks, a barrier island located approximately 3 km off the North Carolina coast, USA. The island is 15 km long and varies between 0.5 and 3 km in width.

There are approximately 14 dominant grass species that vary in distribution along the length of the island (Rubenstein, 1981), though no area hosts species unique to other areas (S. Stuska, personal communication, May 2016). Sandy beaches border the ocean; these are followed by dunes covered primarily with sea oats (*Uniola paniculata*), pennywort (*Hydrocotyle bonariensis*), little bluestem (*Schizachyrium scoparium*), and broomsedge (*Andropogon virginicus*). Flat swales contain patchily distributed grasses including saltmeadow cordgrass (*Spartina patens*), starrush whitetop (*Dichromena colorata*), pennywort, seashore paspalum (*Paspalum vaginatum*), and chairmakers bulrush (*Scirpus americanus*). Maritime forest is dominated by velvet panic grass (*Panicum* sp.), marsh fimbry (*Fimbrystylis spadicea*), and pink muhly grass (*Muhlenbergia capillaris*). Finally, the salt marsh is dominated by smooth cordgrass (*Spartina alterniflora*), salt grass (*Distichlis spicata*), and glasswort (*Salicornia virginica*; Rubenstein & Feinstein, 2021) (M. M. Jones & C. M. V. Nuñez, personal observation).

The island comprises three distinct regions that also vary in habitat visibility (Jones & Nuñez, 2019; Rubenstein, 1981). The western portion of the island contains two primary water sources and is dominated by high dunes and dense brush that limit visibility; animals in this area share resources and exhibit overlapping home ranges. The eastern portion is flat and open, with high visibility; historically, horses in this region were territorial (Rubenstein, 1981), though more recent decreases in available water have necessitated significant home range overlap (Jones & Nuñez, 2019; Marr, 1996). The central portion's terrain is similar to that of the east, facilitating high visibility, though water sources are distributed more evenly, allowing horses to maintain more bounded territories (Jones & Nuñez, 2019, Rubenstein, 1981). Approximately 29%, 32%, and 38% of the animals are located in the western, eastern, and central portions of the island, respectively. The horse population on Shackleford Banks has been comanaged by the National Park Service (NPS) and the Foundation for Shackleford Horses since 1996.

### 2.2 Study subjects

Shackleford horses are typical of feral equids. They form coherent bands of one or sometimes two or three stallion(s) with one to several mare(s) and their offspring (Rubenstein, 1981). Although multi‐male bands can constitute a large portion of feral horse populations, (e.g., 33% in the Kaimanawa horses; Linklater & Cameron, 2000), they occur less frequently on Shackleford Banks, accounting for only 10% and 5% of bands in the 2 years of this study (2015 and 2016, respectively). In addition, differences in the degree of stallion territoriality have been associated with the island's varying ecology (Rubenstein, 1981). During the time of this study, bands in the western portion of the island moved freely within overlapping home ranges, while bands in the central and eastern regions were more likely to defend territories (Jones & Nuñez, 2019). Regardless, bands living in all island regions remained spatially distinct from one another and band membership was easily determined (Feist & McCullough, 1976; Rubenstein, 1981, 1986). Interactions between bands typically involved younger individuals engaging in play and/or exploration, the dispersal of subadult individuals (both male and female), male–male contests, and the transfer of adult females from one band to another (M. M. Jones & C. M. V. Nuñez, unpublished data).

Historically, the bands on Shackleford Banks were stable with most changes to group membership involving the dispersal of immature individuals (Nuñez, 2000; Rubenstein, 1981). Females did leave their males/groups to join others, primarily in response to band stallion quality (Rubenstein, 1994; Rutberg, 1990), but at a low rate (10.8% of females; Rubenstein, 1981). Males sometimes fought to acquire mares from other groups, but stallions almost always retained their mares (Rubenstein, 1981) as is typical in other feral horse populations (Feist & McCullough, 1976). More recently, mares treated with PZP immunocontraception have changed groups more often, making approximately 10 times more group changes than untreated mares and joining (at least temporarily) five times as many groups (Madosky et al., 2010; Nuñez et al., 2009). At the time of this study, 6–7 years after suspension of contraception management, previously treated mares continued to change groups more often than mares that had never been treated (Jones & Nuñez, 2019; Nuñez et al., 2017), providing ample variation in this behavior for comparison.

### 2.3 PZP contraception

The NPS actively administered PZP immunocontraception from 2000 to 2009. During the contraception program, the NPS administered PZP from late February through April each year. Mares received initial and booster doses of PZP; all injections contained 100 μg of PZP plus an adjuvant. Initial doses contained Freund's Complete Adjuvant, Modified, *Mycobacterium butyricum* (Calbiochem #344 289); booster doses contained Freund's Incomplete Adjuvant (Sigma #F5506).

To control for any residual effects of PZP treatment to mare physiology and/or behavior (Nuñez et al., 2017), we considered only mares that had been contracepted at least once between 2000 and 2009 (S. Stuska, personal communication, May 2016); these mares received an average of 4.25 ± 0.28 total treatments (range = 1–7). At the time of this study, PZP treatment had been suspended for approximately 98% of females: only two mares (one mare in 2015; that mare and one additional mare in 2016) actively received PZP treatment (see Table S1). These animals were contracepted primarily due to concerns that they would not survive pregnancy (S. Stuska, personal communication, May 2016).

### 2.4 Animal welfare

All sampling was conducted in accordance with National Research Council standards (National Research Council, 2011). Given the noninvasive nature of this study, neither the Iowa State University's nor the NPS's Institutional Animal Care and Use Committee deemed permitting necessary. This work was conducted under NPS Research Permits CALO‐2015‐0013 and CALO‐2016‐0006.

### 2.5 Behavioral and demographic sampling

Behavioral sampling was conducted primarily by two observers (H.A. Carr, 2015 and M.M. Jones, 2016) and was supplemented with data from four additional observers (M.A.F. Kent and K.E. Monroe, 2015; M. Fatkah and R. Schwartzbeck, 2016). All observers were trained by C.M.V. Nuñez. Comparable to previous studies (Nuñez et al., 2009, 2017; Ransom et al., 2010), data were collected across 8 weeks in 2015 (late June–mid‐August) and 8.5 weeks in 2016 (mid‐June–early August), totaling over 294 h of behavioral observation (192 h, 2015; 102.85 h, 2016), and averaging 3.39 ± 0.20 h per mare (range = 1.23–10.37 h). Horses were identified individually by color, sex, physical condition, and other distinguishing markings, including scars and freeze brands. Ages of the horses are known from long‐term records (Nuñez, 2000; Rubenstein & Nuñez, 2009) and from NPS data (S. Stuska, unpublished data).

We located each study group once every 10 days and once every 3.5 days (on average) in 2015 and 2016, respectively. We recorded each group's GPS location and composition, noting the presence or absence of individual mares. We observed most of the reproductive mares (aged 4 years and older) present during the study, totaling 61 mares, representing 100% and 95% of the potential study population in 2015 and 2016, respectively. Fifty‐four mares were observed in both seasons; the remaining mares were observed in either 2015 (*n* = 4) or 2016 (*n* = 3). Only previously contracepted mares that produced fecal samples (*n* = 30) were considered for these analyses. Ten of the 30 mares that contributed samples changed groups during the study: two mares changed groups in both 2015 and 2016; three mares changed groups in 2015 only; five mares changed groups in 2016 only (see Table S2, Figure S1).

Mare transfer activity was rarely witnessed directly (2015, *n* = 1; 2016, *n* = 1); therefore, mare absence from a band was an important metric with which we measured the number of transfers between groups (Jones & Nuñez, 2019; Nuñez et al., 2009, 2017). We remained with each group for at least 30 min to ensure that individuals recorded as absent were not actually nearby, but out of our sight. Transfer behavior was confirmed by the mares' presence in new bands. In 2015, confirmation was made within 2.50 ± 0.35 days on average (range = 2–3); in 2016, confirmation was made within 13.58 ± 2.49 days on average (range = 1.5–33).

### 2.6 Fecal sampling

Fecal samples were collected opportunistically (Altmann, 1974) during two other investigations addressing (1) the prolonged effects of repeated PZP treatment on mare fertility (in 2015; Nuñez et al., 2017) and (2) stallion behavior and stress physiology (in 2016; Jones et al., 2020; Jones & Nuñez, 2019, 2023). We only collected samples when we were certain of the mares' identity and the samples' location; all samples were still warm at the time of collection. Several fecal balls from each pile were collected and mixed by hand; subsamples (of approximately 0.4 g) were stored in 5 mL of 95% ethanol (Tung et al., 2015). Samples were kept at ambient temperatures for 0.5–4 days until they could be frozen at 80°C.

All observers collected fecal samples. Fifty‐two samples (2015, *n* = 18; 2016, *n* = 34) from 30 PZP‐treated mares were used to assess their microbial communities; we collected 16 samples from mares that changed groups and 36 from mares that did not. Eleven mares produced samples in both 2015 and 2016; the remaining mares produced samples in either 2015 (*n* = 3) or 2016 (*n* = 16; see Table S1). Mares contributed a total of 1.32 ± 0.10 (range = 1–3) and 1.31 ± 0.09 (range = 1–3) samples in 2015 and 2016, respectively. We collected an average of 1.44 samples (2015) and 1.21 samples (2016) per day. On average, 19.28 days (range = 1.5–48.5) elapsed between mare group changing behavior and our subsequent fecal collection (see Table S2).

### 2.7 Body condition

We assessed mare condition via rump scoring as physical condition has been associated with the microbial communities of horses (Garber et al., 2020). We examined the curvature of the line between the tailbone and the point of the hip to determine an individual's rump score. Scores were based on a scale from 1 to 5; a score of 1 being the poorest (Pollock, 1980). Females were scored an average of 1.33 ± 0.14 times (range = 1–2) and 2.08 ± 0.21 times (range = 1–4) in 2015 and 2016, respectively. For analysis, we scaled and centered scores at zero to aid interpretation of results (i.e., the resulting model intercepts can be interpreted as representing mares with an average rump score). For example, the parameter estimate for the intercept of a model of operational taxonomic unit (OTU) richness constructed (using unscaled average rump scores as a predictor variable) reflects the alpha diversity of a horse with a low rump score. Conversely, the intercept of a model constructed using the scaled rump score is representative of horses with an average rump score, a more biologically intuitive approach to model interpretation.

### 2.8 Microbiota analysis

Fecal samples were sent to Argonne National Laboratories for DNA extraction, library preparation, and sequencing. DNA was extracted using the Qiagen DNeasy PowerSoil extraction kit (Qiagen Inc.). The V4 region of the 16S rRNA gene was amplified using the 515F and 806R primers (Caporaso et al., 2012), and barcoded using a primer set adapted for the Illumina MiSeq. Sequence data were generated using paired‐end sequencing for 300 cycles using Illumina MiSeq v2 chemistry. We sequenced 2 × 150 bp amplicons on a MiSeq run, and samples from both years were sequenced in the same run.

Following sequencing, sequence data were demultiplexed, joined, and quality filtered using the Quantitative Insights Into Microbial Ecology (Qiime2) pipeline (version 2‐2018.8; Bolyen et al., 2019). Sequences were trimmed to 150 bp and assigned to suboperational taxonomic units with Deblur (Amir et al., 2017). We assigned taxonomy using the Silva 138 99% OTUs (515F/806R) classifier (Bokulich et al., 2018). A representative sequence for each OTU was aligned with mafft (Katoh et al., 2002) and used to construct a phylogeny with qiime phylogeny (Price et al., 2010). Following preprocessing in Qiime2, the OTU table, phylogenetic tree, taxonomy assignments, and associated metadata for each sample, were imported into R version 4.2.2 (R Core Team, 2022) and converted into a phyloseq object for data exploration and statistical analyses (McMurdie & Holmes, 2013).

### 2.9 Statistical analysis

#### 2.9.1 Alpha diversity

We used linear mixed effects models in R (v.4.2.2) to test whether gut microbiota diversity varied with group transfer behavior (R Core Team, 2022). Prior to analysis of alpha diversity, we subsampled samples to 5706 reads (the number of reads present in the sample with the fewest reads, see Figure S2). Using the subsampled dataset, we calculated three alpha diversity metrics: richness (the number of observed OTUs in each sample), Shannon–Weaver evenness (a measure of the distribution of the relative abundance of each OTU in each sample), and Faith's phylogenetic diversity, which accounts for both the number of OTUs and their phylogenetic relatedness by summing the branch lengths among bacterial taxa represented in an individual's microbial community (Kembel et al., 2010). In our initial maximal models, we tested the effects of group changing behavior, study year, island region, female body condition (average rump score), and one interaction—the interaction between group changing behavior and body condition—on each alpha diversity metric. We tested for this interaction to address the possibility that changes in the microbiota associated with group changing behavior were influenced by mares' physical condition (Garber et al., 2020). All models included a random effect for horse ID to account for nonindependence introduced in situations where a single horse contributed multiple samples. Models were constructed to test all combinations of explanatory variables and were compared with Akaike's Information Criterion adjusted for small sample sizes (AIC_c_; Burnham & Anderson, 2002). We report predictor variables retained in all models with ∆AIC_c_ <2 (Table 1), and parameter estimates for the top models (∆AIC_c_ = 0; Table 2).

**TABLE 1:** Predictor variables retained and model weights of alpha diversity models with ∆AIC_c_ < 2.

Metric	Response variable	Model rank	Predictors	AIC_c_	∆AIC_c_	Model weight
Alpha diversity	Observed richness	1	Group change status × Body condition + Island region + Year	519.50	0.00	0.94
	Evenness	1	Intercept only	−216.70	0.00	0.95
	Faith's phylogenetic diversity	1	Group change status + Island region + Year	242.70	0.00	0.37
		2	Island region + Year	242.90	0.47	0.34

*Note*: All models included a random effect for mare ID.

**TABLE 2:** Parameter estimates for the top alpha diversity models with ∆AIC_c_ = 0.

Diversity metric	Parameter	Value	SE	df	*t*‐Value	*p*‐Value
Observed richness	Intercept	716.34	39.23	28	19.41	.00
	Group change status (Yes)	23.29	26.30	14	0.89	.39
	Body condition	11.12	13.97	14	0.80	.44
	Island region (Mideast)	19.07	37.50	14	0.51	.62
	Island region (West)	−66.64	37.89	28	−1.76	.09
	Year: 2016	−108.39	24.08	14	−4.50	.0005
	Group change status (Yes): Body condition	−20.08	22.26	14	−0.90	.38
Shannon–Weaver evenness	Intercept	0.83	0.00	30	215.26	.00
Faith's phylogenetic diversity	Intercept	41.85	1.39	28	30.15	.00
	Group change status (Yes)	1.04	0.98	16	1.07	.30
	Island region: Mideast	0.19	1.39	16	0.14	.89
	Island region: West	−2.24	1.36	28	−1.65	.11
	Year: 2016	−4.65	0.88	16	−5.31	.0001

*Note*: No metric of alpha diversity (observed richness, Shannon–Weaver evenness, or Faith's phylogenetic diversity) differed between mares with respect to group change status; however, regional and temporal differences were detected.

#### 2.9.2 Beta diversity

##### Betadispersion

Betadispersion is a metric of variability in community composition, in this case, the dissimilarity or distance between an individual mare's microbial community composition and that of its group centroid. Betadispersion values can be used to assess the homogeneity of variance among sample groups (e.g., whether the compositional variance of the microbiota of mares that changed groups was equal to that of mares that did not change groups). We computed betadispersion for Bray–Curtis, UniFrac, and weighted UniFrac metrics using the “betadisper” function in the vegan package (Oksanen et al., 2018). Bray–Curtis dissimilarity reflects how similar (or dissimilar) samples or groups are based on the composition (identity and relative abundance, but not phylogenetic relatedness) of microbial taxa shared between samples. Higher scores in Bray–Curtis space indicate greater dissimilarity (two communities with a score closer to 1 share a smaller proportion of their species than two communities with a score closer to 0). Similar to Bray–Curtis dissimilarities, UniFrac distances account for presence and absence of taxa within communities accounting for phylogenetic relatedness among community members. If two communities differ in Bray–Curtis space but not UniFrac space, it may be because the taxa that differ between communities are closely related, having a high degree of shared branch lengths within the communities. Weighted UniFrac analysis provides a metric of communities' phylogenetic relatedness while accounting for the relative abundance of bacterial taxa, affording a comprehensive survey of the microbiota which is less sensitive to the presence of rare species than presence/absence‐based metrics (Lozupone et al., 2007). Weighted UniFrac distances are quantitative representations of phylogenetic distances (branch lengths) and compositional differences (differences in relative abundances), between communities, with higher values indicating greater distances between communities (Lozupone et al., 2007).

We analyzed differences in betadispersion estimates using the “permutest” function in the vegan package and included a permutation structure to account for nonindependence of samples collected from the same mare. Betadispersion estimates were calculated with respect to median estimates for groups of mares that did or did not change groups.

#### 2.9.3 PERMANOVA

We compared microbial communities between mares that did and did not change groups (controlling for study year, island region, and female body condition) using Bray–Curtis dissimilarities, UniFrac distances, and weighted UniFrac distances calculated using phyloseq, and visualized our comparisons with nonmetric dimensional scaling (NMDS) ordinations in ggplot2 (Wickham, 2016). We conducted permutational analysis of variance (PERMANOVA) with the “adonis2” function (by = “margin”) in the vegan package (Oksanen et al., 2018) to determine whether switching groups had any influence on mares' microbial communities. Our PERMANOVA also included variables to account for microbiota variation due to study year, island region, female body condition, and included a permutation design structure to account for nonindependence of samples collected from the same mare.

### 2.10 Differentially abundant taxa

Finally, we analyzed whether any bacterial families or genera among the OTUs identified were differentially abundant in horse feces in association with several grouping variables including group changing behavior, study year, island region, and female body condition (when appropriate, see below). Estimating differential abundance among taxa in microbiota datasets is notoriously challenging because of the compositional nature of 16S rRNA data, and because differences in sampling fraction, the ratio of absolute abundance of a taxon in a single sample, versus its absolute abundance in the population of samples, can introduce bias and false positives (Lin & Peddada, 2020). We used two methods to assess differentially abundant taxa to accommodate the limitations inherent to each of the differential abundance estimation methods and our study's design. First, we used the more conservative ANCOM‐BC method (Nearing et al., 2022) using the “ancombc” function from the R package ANCOMBC (Lin & Peddada, 2020) which accounts for both compositionality within samples, and differences in sampling fraction among samples. Using this method, we did not assess differentially abundant microbial taxa on the basis of horse condition because organizing this continuous metric into biologically relevant groups would have yielded unacceptably small sample sizes per group. Prior to testing for differential abundance with ANCOM‐BC, we removed taxa observed in fewer than 10% of samples to avoid any undue influence of rare taxa in our analysis. We then analyzed differential abundance on raw abundance data. Next, we used the MaAsLin2 method from the Maaslin2 R package (Mallick et al., 2021) which other researchers have noted to be less conservative in comparison with ANCOM‐BC, but highly consistent (Nearing et al., 2022), to allow for the inclusion of continuous predictor variables and random effects. Using this method, we were able to assess differentially abundant microbial taxa based on mare body condition. Additionally, we were able to include a random effect to account for instances in which multiple samples were collected from the same mares. We tested for differentially abundant taxa at the genus and family levels, again filtering out taxa that were present in fewer than 10% of samples, and whose relative abundances were <0.01%. Differential abundance analysis in MaAsLin2 was performed on raw (non‐rarefied) count data, and data were transformed with the default log transformation.

## 3 RESULTS

### 3.1 Alpha diversity

**FIGURE 1:**
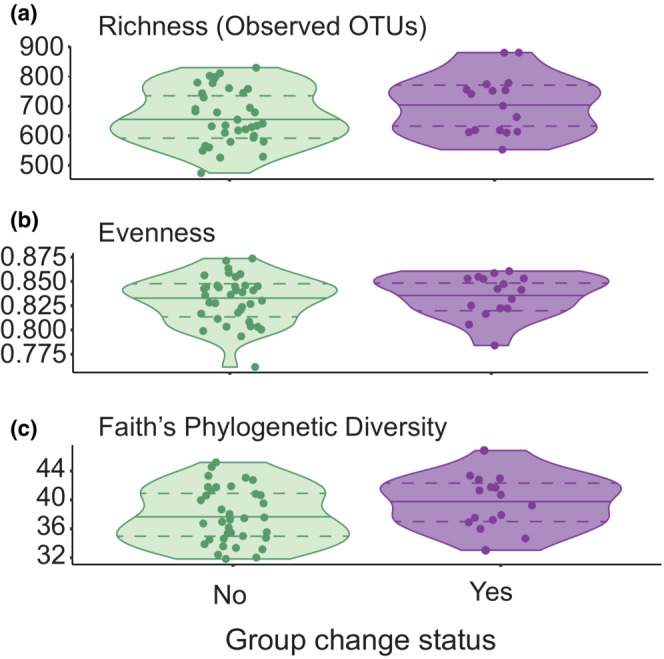
Group changing status of Shackleford Banks, NC mares was not associated with microbiota alpha diversity in terms of its richness (a), evenness (b), or phylogenetic diversity (c). Each point represents a single fecal sample; some mares produced multiple samples. Median alpha diversity for each metric is shown with a solid line, while upper and lower dashed lines represent the 75th and 25th quantiles, respectively.

Group‐changing behavior was not significantly associated with fecal microbiota richness, evenness, or phylogenetic diversity in mares (*p*_obs_ = .333, *p*_even_ = NA, *p*_PPD_ = .257; Figure 1). Observed richness was best described by one model; Shannon–Weaver evenness was best described by an intercept‐only model; and Faith's phylogenetic diversity was described by two models with ∆AIC_c_ < 2. We report the parameter estimates, standard errors, and *p*‐values of the top models (∆AIC_c_ = 0) in Table 2.

Alpha diversity (calculated using richness and phylogenetic diversity metrics) was significantly lower in 2016 than in 2015 (*p*_obs_ < .0001, *p*_PD_ < .001). Additionally, samples collected from horses in the western region of the island tended to be less rich than samples from horses in the eastern and central regions of the island (*p*_obs_ = .09). Phylogenetic diversity of western samples was also somewhat reduced in comparison to samples from other regions (*p*_PD_ = .03, .11). No variation in community evenness was observed across years, regions, body condition, or group changing behavior status.

### 3.2 Beta diversity

**FIGURE 2:**
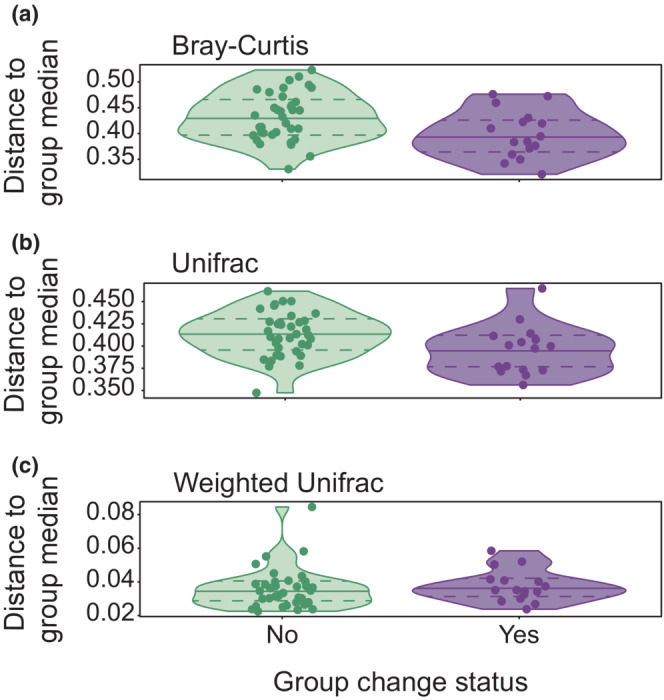
Betadispersion estimates differed between Shackleford Banks, NC mares that changed social groups and those that did not in Bray–Curtis space (a) and unweighted UniFrac space (b) but not in weighted UniFrac space (c). Each point represents a single fecal sample; some mares produced multiple samples. Median alpha diversity for each metric is shown with a solid line, while upper and lower dashed lines represent the 75th and 25th quantiles, respectively.

Betadispersion estimates for mares that changed groups were higher in both UniFrac and Bray–Curtis space (*p* = .002), but this pattern was not repeated with weighted UniFrac distances (Table 3, Figure 2).

**TABLE 3:** Betadispersion, or within‐group variation in beta diversity, differed between mares that did and did not change groups when compared in Bray–Curtis and unweighted UniFrac space but did not differ between groups in weighted UniFrac space.

Diversity metric	df	Sum sq	Mean sq	*F*	N perm	*p*‐Value
Bray–Curtis						
Groups	1	0.01	0.01	6.16	499	.002
Residuals	50	0.11	0.00	–	–	–
UniFrac						
Groups	1	0.00	0.00	5.43	499	.0002
Residuals	50	0.04	0.00	–	–	–
Weighted UniFrac						
Groups	1	0.00	0.00	0.29	499	.65
Residuals	50	0.01	0.00	–	–	–

*Note*: All models included a permutation structure to account for multiple samples being collected from the same mare.

**FIGURE 3:**
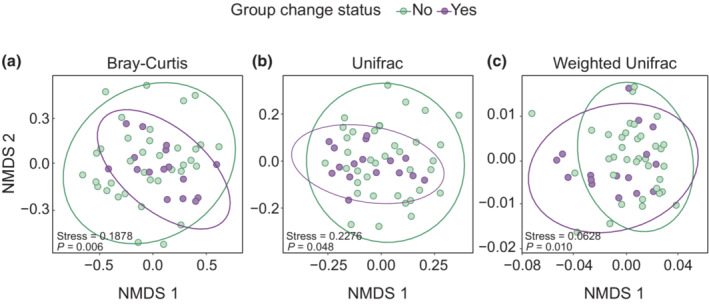
Group changing behavior by Shackleford Banks, NC mares was associated with a shift in microbial community composition, as described by Bray–Curtis dissimilarities (a) and unweighted (b) and weighted UniFrac (c) distances. Each point represents a single fecal sample; some mares produced multiple samples. Stress values refer to the stress of the ordination solution and printed *p*‐values are derived from PERMANOVA analysis describing the contribution of group‐change status to microbiota variation (reported in Table 4).

In Bray–Curtis, UniFrac, and weighted UniFrac spaces, mares' fecal microbial communities were significantly different from each other based on group changing behavior even after controlling for the effects of study year, island region, female body condition, and repeated measurements of several individual mares (*p*_all_ < .05; Table 4, Figure 3). Using all metrics of beta diversity, fecal bacterial communities also varied by year (*p* < .01; Table 4). Additionally, there was a significant association between female body condition and community composition in weighted UniFrac space (*p* = .008; Table 4).

**TABLE 4:** PERMANOVA analysis.

Diversity metric	Term	df	Sum sq	*R* ^2^	*F*	*p*‐Value
Bray–Curtis	Year	1	0.57	0.07	3.55	.004
	Island region	2	0.64	0.07	1.97	.30
	Body condition	1	0.24	0.03	1.47	.13
	Group change status	1	0.22	0.02	1.35	.006
	Residual	13	6.92	0.80	–	–
	Total	48	8.69	1.00	–	–
UniFrac	Year	1	0.47	0.06	3.03	.002
	Island region	2	0.58	0.07	1.89	.36
	Body condition	1	0.18	0.02	1.19	.74
	Group change status	1	0.19	0.02	1.23	.04
	Residual	43	6.61	0.82	–	–
	Total	48	8.08	1.00	–	–
Weighted UniFrac	Year	1	0.01	0.14	9.43	.004
	Island region	2	0.00	0.05	1.73	.27
	Body condition	1	0.00	0.04	2.46	.008
	Group change status	1	0.00	0.04	2.41	.01
	Residual	43	0.04	0.66	–	–
	Total	48	0.07	1.00	–	–

*Note*: All model terms were assessed for marginal significance and all models included a permutation structure to account for repeated sampling of the same mare.

### 3.3 Differentially abundant taxa

Prior to running differential abundance analyses with ANCOM‐BC and MaAsLin2, we filtered count tables to retain only families and genera present in >10% of samples which yielded a family‐level count table with 69 families, and a genus‐level count table with 136 genera. We considered families or genera to be differentially abundant if their presence in a focal group (mares that changed groups, mares from a particular island region) was associated with an FDR‐corrected *p*‐value (*q*‐value) <.05.

#### 3.3.1 Differentially abundant bacterial families

No bacterial families were shown to be differentially abundant in association with group changing behavior using ANCOM‐BC, while MaAsLin2 identified two bacterial families, family Eubacteriaceae and an unclassified family (001) within the order Bacteroidales which were more abundant in feces of mares that changed groups (Table 5).

**TABLE 5:** Differentially abundant bacterial families and genera detected in mare fecal samples from mares that changed groups versus those that did not, organized by method used for differential abundance analysis, and level of taxonomic identity (family or genus) and from most differentially absent (detected less than expected by chance) to differentially abundant (detected more than expected by chance).

Taxonomic level	Method	Taxa ID	Log fold change	Beta	SE	*p*‐Value	*q*‐Value
Family	ANCOM‐BC	–	–	–	–	–	–
	MaAsLin2	Eubacteriaceae	–	0.64	0.19	.001783	0.030755
		Bacteroidales_UCG.001	–	1.27	0.40	.002641	0.043384
Genus	ANCOM‐BC	*Blautia*	−0.80	–	0.24	9.78e‐04	0.026624
		Lachnospiraceae NK4A136 group	0.45	–	0.13	5.24e‐04	0.023789
		*Caldicoprobacter*	0.78	–	0.19	3.12e‐05	0.004241
		Uncultured genus in Family: Marinifilaceae	0.82	–	0.24	7.35e‐04	0.025004
		p‐1088‐a5 gut group	0.95	–	0.27	3.74e‐04	0.023789
	MaAsLin2	*Blautia*	–	−1.90	0.54	.001211	0.032267
		*Eubacterium*	–	0.64	0.19	.001782	0.013247
		Uncultured genus in Family: Erysipelotrichaceae	–	1.14	0.29	.000390	0.013246

#### 3.3.2 Differentially abundant bacterial genera

**FIGURE 4:**
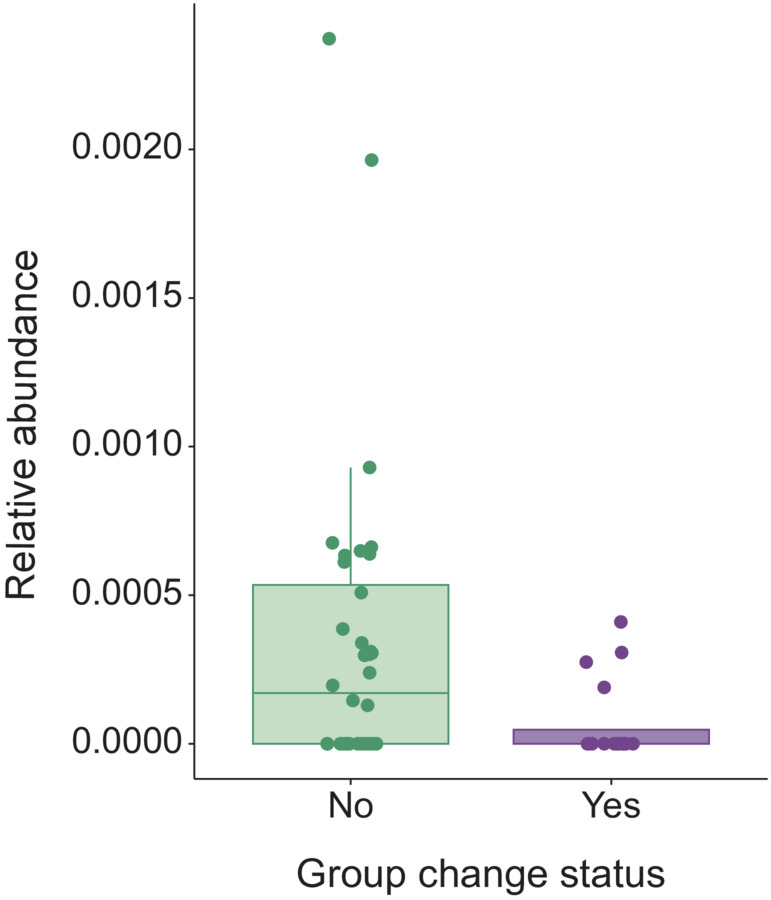
Bacteria in the genus *Blautia* (Phylum: Firmicutes, Class: Clostridia) were present in 24 of 52 fecal samples collected from Shackleford Banks, NC mares. The bacteria were significantly less abundant in the feces of mares that changed groups than in the feces of those that did not (FDR‐corrected *p* < .05).

Differential abundance analysis with ANCOM‐BC identified four bacterial genera: an uncultured genus in the family Marinifilaceae, *Caldicoprobacter* (order Clostridiales, phylum Firmicutes), the NK4A136 group in the genus *Lachnospiraceae*, and an unnamed genus (p‐1088‐a5_gut_group) in family Pirellulaceae, that were significantly more abundant and one genus (*Blautia*) that was differentially less abundant in the feces of horses that changed groups (Table 5). Using MaAsLin2, we identified two genera, *Eubacterium* and an uncultured genus in family Erysipelotrichaceae that were significantly more abundant in feces from mares that changed groups, and one genus (*Blautia*) for which abundance was lower in mares that changed groups (Table 5). Only the bacterial genus *Blautia* was identified by both methods of differential abundance analysis as varying with group changing behavior (Figure 4).

#### 3.3.3 Associations with body condition, year, and region

Neither method of analysis identified differential abundances of taxa at the family or genus level with mare body condition. In contrast, both methods identified differential abundances of several taxa (at both the family and genus level) with study year and region: we detected nine differentially abundant families and seven differentially abundant genera associated with study year, and three differentially abundant families and two differentially abundant genera associated with study region (Tables S3 and S4).

## 4 DISCUSSION

Here, we show that fecal microbiota composition was associated with group changing behavior (a proxy for social instability) in feral mares. Contrary to our predictions, no metric of alpha diversity, including richness, evenness, and phylogenetic diversity, differed for mares that changed groups compared to mares that did not. However, the composition of the gut microbiota of mares that changed groups was distinct from those of mares that did not in Bray–Curtis, UniFrac, and weighted UniFrac spaces. These results indicate that there is a small (*R* ^2^ = .025, .038) but significant (*p* < .05) difference between the composition of mares' fecal microbial communities that do and do not change groups.

We observed differences between communities using PERMANOVA, which is intended to detect significant shifts in group centroids, indicative of among‐group variation. However, this type of analysis can be confounded by large differences in within‐group variation. To address this possibility, we also compared betadispersion estimates among groups of mares and found differences in Bray–Curtis and UniFrac spaces associated with group changing behavior. Compositional differences in Bray–Curtis and UniFrac spaces could be driven by the heterogeneity of variances within communities, by differences in community membership, or by phylogenetic relatedness to varying levels. If greater within‐group variation among mares that changed bands drives the community‐level differences detected with PERMANOVA, it would suggest that social instability may precipitate microbiota community instability. However, our results in weighted UniFrac space provide more conclusive evidence that bacterial communities of mares that did and did not change groups are compositionally different. Specifically, in weighted UniFrac space, the difference we observed between‐group centroids was not accompanied by marked differences in within‐group variation between mares that did and did not change social groups. Rather, our analyses in weighted UniFrac space provide evidence of a more directional shift in the composition of the fecal microbiota community. Taken together, our results suggest that social perturbations (like group changing behavior) may not precipitate wholesale shifts in bacterial diversity as measured by richness or relatedness (alpha diversity). Instead, social perturbations may initiate more subtle shifts in community structure. These changes are driven by differing levels of intragroup variation (depending on the metric used to compare communities), as well as combinations of changes in the relative abundances of bacterial taxa, the identities of taxa within communities, and their relatedness within communities (beta diversity). Although our study does not address the consequences of such a shift, we argue that detecting these shifts and considering their possible mechanistic origins may shed light on the cascading effects of social perturbation in group‐living animals.

There are myriad mechanisms that could be driving these changes. For example, shifts in microbial community composition are often linked to changes in diet (Amato et al., 2013; Ley et al., 2006, 2008; Turnbaugh et al., 2009; Wu et al., 2011; Yildirim et al., 2010). In our study, dietary changes (including changes to both forage and water sources) and/or increased microbial transfer between newly encountered individuals (concomitant with changes in home range and/or region occurring after successful group switching behavior) likely played an important role in the changes to mares' fecal microbial communities (Stothart et al., 2021). Feral horse home ranges and group composition are typically stable (Berger, 1977; Feist & McCullough, 1976; Klingel, 1975; Linklater et al., 2000; Rubenstein, 1981), resulting in more consistent diets and environmental microbiota, thereby driving microbiota similarity among group members (Antwis et al., 2018; Stothart et al., 2021). Increased dispersal between communities is thought to stabilize populations (Crowley, 1981); however, mares that change groups repeatedly may not remain in groups long enough to develop microbial communities that are representative of any one location (region or home range), instead experiencing increased exposure to bacterial communities from new environments and new group members. Work with semi‐feral Welsh ponies has demonstrated that microbiota communities can vary with spatial structuring and even with specific social interactions within bands (Antwis et al., 2018). Similarly, mares that change groups likely experience transitional fecal microbial communities from one band to the other, depending on how long they remain with each band; though again, these communities may not be representative of any one location. Such “transitional” microbiota communities have been demonstrated in dispersing male baboons: immigrant males that had lived in their current social group for a shorter period had microbiota that were less similar to other long‐term group residents than did males with longer group residency times (Grieneisen et al., 2017). Such “transitional” microbial communities may leave mares that change groups (and particularly mares engaging in this behavior more often) more susceptible to environmental shifts; whether they constitute alterations to the surrounding environment (due to drought, hurricanes, increased/decreased temperatures, etc.) or to the hosts' own physiology (due to reproductive state, group membership, physical condition, etc.; McKenzie et al., 2017). We did not have sufficient sample sizes to test for such effects in this study; future work should capitalize on these transitions and monitor their potential effects.

The strong association between year and mares' fecal microbial communities may indicate several changes over the course of the study, including ecological differences, shifts in diet, and/or variable social dynamics. Mean and maximum temperatures during the study were similar between years (Table 6). Mean precipitation was slightly higher in 2015 (Table 6), though it is unlikely that this relatively small difference alone would have accounted for the stark differences in mare microbiota communities across years. While these conditions could indicate similar forage availability across years, shifts in diet cannot be ruled out. That said, even subtle differences in sociality have been shown to affect feral horse microbial community composition. As previously described, studies with semi‐feral Welsh ponies have demonstrated more similar fecal microbial communities among bands and even among more closely associated individuals within bands, indicating the importance of bacterial dispersal among close associates (Antwis et al., 2018). Work with the feral horses of Sable Island, Nova Scotia, demonstrated similar effects and revealed the importance of parental status (among mares) and ecological drift to feral horse microbiota communities (Stothart et al., 2021). It is possible that any one or several of these nonmutually exclusive effects could have influenced mares' microbiota communities differentially between years.

**TABLE 6:** Mean and maximum temperature, mean precipitation (±standard error) during the study (National Climate Data Center, 2022).

Year	Mean temperature	Mean max. temperature	Mean precipitation
2015	26.26°C ± 0.02	29.49°C ± 0.02	5.19 cm ± 0.14
2016	26.90°C ± 0.02	30.15°C ± 0.01	3.65 cm ± 0.01

Still, even after accounting for year, island region, and mare body condition, the common act of changing groups was associated with changes to the microbial community, indicating that the social perturbation itself was associated with at least some of the changes to mares' microbiota communities. Group changing behavior can induce stress responses in Shackleford mares (Nuñez et al., 2014). Mares changing groups are often chased, and sometimes kicked and/or bitten as resident stallions attempt to herd them back to the group (Madosky et al., 2010); they are subject to increased levels of reproductive behavior by both resident and new stallions (Nuñez et al., 2009); and often find themselves near highly escalated male–male conflicts (Jones & Nuñez, 2019). Moreover, mares making successful changes to new bands are frequently subject to aggression from resident females (C. M. V. Nuñez, unpublished data). Though we cannot confirm that the differences exhibited here were due to mare stress physiology, chronic or prolonged stressors affect the microbial communities of diverse taxa in both the laboratory and in the wild. Mice, rhesus monkeys, red squirrels, yellow‐legged gulls, and corals all show changes to their microbiota with increased stress (Bailey et al., 2010, 2011; Bailey & Coe, 1999; Noguera et al., 2018; Stothart et al., 2016; Zaneveld et al., 2016). Most relevant to this study, are the effects of transport, weaning, and colitis infection on the microbiota of domestic horses (Costa et al., 2012; Mach et al., 2017; Schoster et al., 2016). Myriad changes have been documented; however, decreases in the relative abundance of bacteria in order Clostridiales and increases in the abundance of bacteria in genus *Bacteroides* are notable (Costa et al., 2012, Mach et al., 2017, Schoster et al., 2016).

Interestingly, we found a pattern of consistent depletion of bacteria in the *Blautia* genus among mares that changed groups. The *Blautia* genus includes several bacteria that were previously classified in the genera *Clostridium* and *Ruminococcus* (Liu et al., 2021). Bacteria in this genus are strictly anaerobic and some strains of this genus have been associated with probiotic activity and anti‐inflammatory activity (Liu et al., 2021). In captive horses, increased abundance of *Blautia* bacteria has been associated with an obesity phenotype (Biddle et al., 2018), while researchers studying human subjects in Japan noted a strong negative relationship between *Blautia* relative abundance and visceral fat accumulation (Ozato et al., 2019). Our study did not include measurements of diet or metabolism and draws no links between *Blautia* abundance and metrics of mare health or body condition. Despite this, given the strong associations between bacteria in the *Blautia* genus with host diet and metabolism, and the previously discussed likelihood that group changing behavior may entail concomitant shifts in diet, our observation of a consistent shift in *Blautia* abundance offers an intriguing glimpse into a possible mechanism by which a social perturbation (group changing) may effect changes in the gut microbiota.

Overall, our findings suggest that although mares' group changing behavior is not associated with the number of bacterial taxa present in their fecal microbial communities, it may account for differences in the relative abundances, taxonomic identities, and relatedness of bacterial taxa, with group changing mares showing a shift away from the typical (non‐changer) microbial community. Regardless of their exact origin(s) (social disruption and its associated behavioral and physiological effects, dietary changes, increased microbial transfer with new individuals, etc.), these changes could prove critical to herbivorous animals that are heavily dependent upon their microbiota for nutrient absorption. Still, whether the changes to the mares' fecal microbial communities with group changing behavior are beneficial, detrimental, or neutral cannot be definitively determined without direct manipulation. Moreover, we cannot determine whether mares changing groups demonstrate changes to their microbial communities because of their behavior or if they harbor communities different from those of mares that stay with their groups from the outset. Only by sampling mares before and after group changing behavior can we better understand this dynamic. Unfortunately, the logistics of field work and the serendipity of fecal collection did not afford us with ample sample sizes to perform this analysis (before samples *n* = 8; after samples *n* = 7). In addition, investigating the ways in which other factors, like helminth levels (Koch & Schmid‐Hempel, 2011; Zaiss & Harris, 2016) and immune responses (Macpherson et al., 2018) may correlate with changes to mares' microbiota communities will be important if we are to better understand the potential mechanisms of such alterations.

Taken together, our results link social disruption with community‐level shifts to the bacterial communities of a highly social and microbiota‐dependent mammal, the feral horse. Although correlations between social interactions and the microbiota have been demonstrated in the wild (Antwis et al., 2018; Stothart et al., 2021), our research suggests an association between specific social perturbations and the microbiota, further elucidating the myriad links between animal behavior and physiology. Moreover, our results could have important management implications. The behavioral and physiological changes demonstrated by contracepted mares highlight the potential for management strategies to have unexpected and far‐reaching implications for wildlife. Shackleford mares treated with PZP change groups more often and join more groups than do untreated mares (Madosky et al., 2010; Nuñez et al., 2009); these behavioral effects can be long‐lasting, even after treatment has ended (Nuñez et al., 2017). PZP is used to control animal populations around the world, including white‐tailed deer (*Odocoileus virginianus*; McShea et al., 1997), elk (*Cervus canadensis*; Heilmann et al., 1998), the endangered African elephant (*Loxodonta africana*; Bertschinger et al., 2018), and Przewalski's horse (*Equus ferus przewalskii*; Kerekes et al., 2021). Our previous work on Shackleford has shown that fewer contraceptive treatments, over longer periods of time can help to maintain more natural behavior and reproductive physiology (Nuñez et al., 2017) and reduce stress (Nuñez et al., 2014); given the present data, such management practice could also potentially maintain important symbiotic relationships in treated mares. Whether such relationships hold in other managed wildlife seems a worthwhile focus for future study.

## AUTHOR CONTRIBUTIONS

**Grace J. Vaziri:** Data curation (equal); formal analysis (lead); visualization (lead); writing – original draft (equal); writing – review and editing (equal). **Maggie M. Jones:** Investigation (equal); methodology (equal). **Haley A. Carr:** Investigation (equal). **Cassandra M. V. Nuñez:** Conceptualization (lead); data curation (equal); formal analysis (supporting); funding acquisition (lead); investigation (supporting); methodology (equal); project administration (lead); resources (lead); supervision (lead); visualization (supporting); writing – original draft (equal); writing – review and editing (equal).

## Supporting information


Figure S1

Figure S2

Table S1

Table S2

Table S3

Table S4

## Data Availability

*Genetic data*: Sequence data are archived at the NCBI BioProject ID PRJNA962045 and can be accessed with the following link: https://www.ncbi.nlm.nih.gov/bioproject/962045. *Sample metadata*: Metadata are available on the Open Science Framework data repository (Vaziri et al., 2022) at the following link: https://osf.io/rhz7a/.

## References

[ece310079-bib-0001] Altmann, J. (1974). Observational study of behavior: Sampling methods. Behaviour, 49, 227–267. 4597405 10.1163/156853974x00534

[ece310079-bib-0002] Amato, K. R. , Yeoman, C. J. , Kent, A. , Righini, N. , Carbonero, F. , Estrada, A. , Rex Gaskins, H. , Stumpf, R. M. , Yildirim, S. , Torralba, M. , Gillis, M. , Wilson, B. A. , Nelson, K. E. , White, B. A. , & Leigh, S. R. (2013). Habitat degradation impacts black howler monkey (*Alouatta pigra*) gastrointestinal microbiomes. The ISME Journal, 7, 1344–1353. 23486247 10.1038/ismej.2013.16 PMC3695285

[ece310079-bib-0003] Antwis, R. E. , Lea, J. M. D. , Unwin, B. , & Shultz, S. (2018). Gut microbiome composition is associated with spatial structuring and social interactions in semi‐feral Welsh Mountain ponies. Microbiome, 6, 207. 30466491 10.1186/s40168-018-0593-2 PMC6251106

[ece310079-bib-0004] Bailey, M. T. (2012). The contributing role of the intestinal microbiota in stressor‐induced increases in susceptibility to enteric infection and systemic immunomodulation. Hormones and Behavior, 62, 286–294. 22366706 10.1016/j.yhbeh.2012.02.006

[ece310079-bib-0005] Bailey, M. T. , & Coe, C. L. (1999). Maternal separation disrupts the integrity of the intestinal microflora in infant rhesus monkeys. Developmental Psychobiology, 35, 146–155. 10461128

[ece310079-bib-0006] Bailey, M. T. , Dowd, S. E. , Galley, J. D. , Hufnagle, A. R. , Allen, R. G. , & Lyte, M. (2011). Exposure to a social stressor alters the structure of the intestinal microbiota: Implications for stressor‐induced immunomodulation. Brain, Behavior, and Immunity, 25, 397–407. 21040780 10.1016/j.bbi.2010.10.023 PMC3039072

[ece310079-bib-0007] Bailey, M. T. , Dowd, S. E. , Parry, N. M. , Galley, J. D. , Schauer, D. B. , & Lyte, M. (2010). Stressor exposure disrupts commensal microbial populations in the intestines and leads to increased colonization by *Citrobacter rodentium* . Infection and Immunity, 78, 1509–1519. 20145094 10.1128/IAI.00862-09 PMC2849416

[ece310079-bib-0008] Berger, J. (1977). Organizational systems and dominance in feral horses in Grand Canyon. Behavioral Ecology and Sociobiology, 2, 131–146.

[ece310079-bib-0009] Bertschinger, H. J. , Delsink, A. , Van Altena, J. , & Kirkpatrick, J. F. (2018). Porcine zona pellucida vaccine immunocontraception of African elephant (*Loxodonta africana*) cows: A review of 22 years of research. Bothalia‐African Biodiversity & Conservation, 48, 1–8.

[ece310079-bib-0010] Biddle, A. S. , Tomb, J.‐F. , & Fan, Z. (2018). Microbiome and blood analyte differences point to community and metabolic signatures in lean and obese horses. Frontiers in Veterinary Science, 5, 225. 30294603 10.3389/fvets.2018.00225 PMC6158370

[ece310079-bib-0011] Bokulich, N. A. , Kaehler, B. D. , Rideout, J. R. , Dillon, M. , Bolyen, E. , Knight, R. , Huttley, G. A. , & Gregory Caporaso, J. (2018). Optimizing taxonomic classification of marker‐gene amplicon sequences with QIIME 2's q2‐feature‐classifier plugin. Microbiome, 6, 90. 29773078 10.1186/s40168-018-0470-z PMC5956843

[ece310079-bib-0012] Bolyen, E. , Rideout, J. R. , Dillon, M. R. , Bokulich, N. A. , Abnet, C. C. , Al‐Ghalith, G. A. , Alexander, H. , Alm, E. J. , Arumugam, M. , & Asnicar, F. (2019). Reproducible, interactive, scalable and extensible microbiome data science using QIIME 2. Nature Biotechnology, 37, 852–857. 10.1038/s41587-019-0209-9 PMC7015180 31341288

[ece310079-bib-0013] Burnham, K. P. , & Anderson, D. R. (2002). Model selection and multimodel inference: A practical information‐theoretic approach (2nd ed.). Springer‐Verlag.

[ece310079-bib-0014] Caporaso, J. G. , Lauber, C. L. , Walters, W. A. , Berg‐Lyons, D. , Huntley, J. , Fierer, N. , Owens, S. M. , Betley, J. , Fraser, L. , Bauer, M. , Gormley, N. , Gilbert, J. A. , Smith, G. , & Knight, R. (2012). Ultra‐high‐throughput microbial community analysis on the Illumina HiSeq and MiSeq platforms. The ISME Journal, 6, 1621–1624. 22402401 10.1038/ismej.2012.8 PMC3400413

[ece310079-bib-0015] Comizzoli, P. , Power, M. L. , Bornbusch, S. L. , & Muletz‐Wolz, C. R. (2021). Interactions between reproductive biology and microbiomes in wild animal species. Animal Microbiome, 3, 87. 34949226 10.1186/s42523-021-00156-7 PMC8697499

[ece310079-bib-0016] Costa, M. C. , Arroyo, L. G. , Allen‐Vercoe, E. , Stämpfli, H. R. , Kim, P. T. , Sturgeon, A. , & Weese, J. S. (2012). Comparison of the fecal microbiota of healthy horses and horses with colitis by high throughput sequencing of the V3‐V5 region of the 16S rRNA gene. PLoS One, 7, e41484. 22859989 10.1371/journal.pone.0041484 PMC3409227

[ece310079-bib-0017] Crowley, P. H. (1981). Dispersal and the stability of predator‐prey interactions. The American Naturalist, 118, 673–701.

[ece310079-bib-0018] Feist, J. D. , & McCullough, D. R. (1976). Behavior patterns and communication in feral horses. Zeitschrift für Tierpsychologie, 41, 337–371. 983427 10.1111/j.1439-0310.1976.tb00947.x

[ece310079-bib-0019] Garber, A. , Hastie, P. , & Murray, J.‐A. (2020). Factors influencing equine gut microbiota: Current knowledge. Journal of Equine Veterinary Science, 88, 102943. 32303307 10.1016/j.jevs.2020.102943

[ece310079-bib-0020] Grieneisen, L. E. , Livermore, J. , Alberts, S. , Tung, J. , & Archie, E. A. (2017). Group living and male dispersal predict the core gut microbiome in wild baboons. Integrative and Comparative Biology, 57, 770–785. 29048537 10.1093/icb/icx046 PMC5886331

[ece310079-bib-0021] Heilmann, T. J. , Garrott, R. A. , Cadwell, L. L. , & Tiller, B. L. (1998). Behavioral response of free‐ranging elk treated with an immunocontraceptive vaccine. Journal of Wildlife Management, 62, 243–250.

[ece310079-bib-0022] Jones, M. M. , & Nuñez, C. (2023). Laissez‐faire stallions? Males' fecal cortisol metabolite concentrations do not vary with increased female turnover in feral horses (*Equus caballus*). Animals, 13, 176. 36611784 10.3390/ani13010176 PMC9817692

[ece310079-bib-0023] Jones, M. M. , & Nuñez, C. M. V. (2019). Decreased female fidelity alters male behavior in a feral horse population managed with immunocontraception. Applied Animal Behaviour Science, 214, 34–41.

[ece310079-bib-0024] Jones, M. M. , Proops, L. , & Nuñez, C. M. V. (2020). Rising up to the challenge of their rivals: Mare infidelity intensifies stallion response to playback of aggressive conspecific vocalizations. Applied Animal Behaviour Science, 225, 104949.

[ece310079-bib-0025] Kaseda, Y. , Khalil, A. M. , & Ogawa, H. (1995). Harem stability and reproductive success of Misaki feral mares. Equine Veterinary Journal, 27, 368–372. 8654352 10.1111/j.2042-3306.1995.tb04072.x

[ece310079-bib-0026] Katoh, K. , Misawa, K. , Kuma, K. I. , & Miyata, T. (2002). MAFFT: A novel method for rapid multiple sequence alignment based on fast Fourier transform. Nucleic Acids Research, 30, 3059–3066. 12136088 10.1093/nar/gkf436 PMC135756

[ece310079-bib-0027] Kembel, S. W. , Cowan, P. D. , Helmus, M. R. , Cornwell, W. K. , Morlon, H. , Ackerly, D. D. , Blomberg, S. P. , & Webb, C. O. (2010). Picante: R tools for integrating phylogenies and ecology. Bioinformatics, 26, 1463–1464. 20395285 10.1093/bioinformatics/btq166

[ece310079-bib-0028] Kerekes, V. , Sándor, I. , Nagy, D. , Ozogány, K. , Göczi, L. , Ibler, B. , Széles, L. , & Barta, Z. (2021). Trends in demography, genetics, and social structure of Przewalski's horses in the Hortobagy National Park, Hungary over the last 22 years. Global Ecology and Conservation, 25, e01407.

[ece310079-bib-0029] Klingel, H. (1975). Social organization and reproduction in equids. Journal of Reproduction and Fertility, 23, 7–11. 1060868

[ece310079-bib-0030] Koch, H. , & Schmid‐Hempel, P. (2011). Socially transmitted gut microbiota protect bumble bees against an intestinal parasite. Proceedings of the National Academy of Sciences of the United States of America, 108, 19288–19292. 22084077 10.1073/pnas.1110474108 PMC3228419

[ece310079-bib-0031] Ley, R. E. , Hamady, M. , Lozupone, C. , Turnbaugh, P. J. , Ramey, R. R. , Bircher, J. S. , Schlegel, M. L. , Tucker, T. A. , Schrenzel, M. D. , & Knight, R. (2008). Evolution of mammals and their gut microbes. Science, 320, 1647–1651. 18497261 10.1126/science.1155725 PMC2649005

[ece310079-bib-0032] Ley, R. E. , Turnbaugh, P. J. , Klein, S. , & Gordon, J. I. (2006). Human gut microbes associated with obesity. Nature, 444, 1022–1023. 17183309 10.1038/4441022a

[ece310079-bib-0033] Lin, H. , & Peddada, S. D. (2020). Analysis of compositions of microbiomes with bias correction. Nature Communications, 11, 1–11. 10.1038/s41467-020-17041-7 PMC7360769 32665548

[ece310079-bib-0034] Linklater, W. L. , & Cameron, E. Z. (2000). Tests for cooperative behaviour between stallions. Animal Behaviour, 60, 731–743. 11124871 10.1006/anbe.2000.1525

[ece310079-bib-0035] Linklater, W. L. , Cameron, E. Z. , Minot, E. O. , & Stafford, K. J. (1999). Stallion harassment and the mating system of horses. Animal Behaviour, 58, 295–306. 10458881 10.1006/anbe.1999.1155

[ece310079-bib-0036] Linklater, W. L. , Cameron, E. Z. , Stafford, K. J. , & Veltman, C. J. (2000). Social and spatial structure and range use by Kaimanawa wild horses (*Equus caballus*: Equidae). New Zealand Journal of Ecology, 24, 139–152.

[ece310079-bib-0037] Liu, X. , Mao, B. , Gu, J. , Wu, J. , Cui, S. , Wang, G. , Zhao, J. , Zhang, H. , & Chen, W. (2021). Blautia—A new functional genus with potential probiotic properties? Gut Microbes, 13, 1–21. 10.1080/19490976.2021.1875796 PMC7872077 33525961

[ece310079-bib-0038] Lozupone, C. A. , Hamady, M. , Kelley, S. T. , & Knight, R. (2007). Quantitative and qualitative β diversity measures lead to different insights into factors that structure microbial communities. Applied Environmental Microbiology, 73, 1576–1585. 17220268 10.1128/AEM.01996-06 PMC1828774

[ece310079-bib-0039] Mach, N. , Foury, A. , Kittelmann, S. , Reigner, F. , Moroldo, M. , Ballester, M. , Esquerré, D. , Rivière, J. , Sallé, G. , Gérard, P. , Moisan, M.‐P. , & Lansade, L. (2017). The effects of weaning methods on gut microbiota composition and horse physiology. Frontiers in Physiology, 8, 535. 28790932 10.3389/fphys.2017.00535 PMC5524898

[ece310079-bib-0040] Macpherson, A. J. , Yilmaz, B. , Limenitakis, J. P. , & Ganal‐Vonarburg, S. C. (2018). IgA function in relation to the intestinal microbiota. Annual Review of Immunology, 36, 359–381. 10.1146/annurev-immunol-042617-053238 29400985

[ece310079-bib-0041] Madosky, J. M. (2011). *Factors that affect harem stability in a feral horse (*Equus caballus*) population on Shackleford Banks Island, NC* . University of New Orleans, New Orleans, LA, USA. http://search.proquest.com/pqdtft/advanced?accountid=13314

[ece310079-bib-0042] Madosky, J. M. , Rubenstein, D. I. , Howard, J. J. , & Stuska, S. (2010). The effects of immunocontraception on harem fidelity in a feral horse (*Equus caballus*) population. Applied Animal Behaviour Science, 128, 50–56.

[ece310079-bib-0043] Mallick, H. , Rahnavard, A. , McIver, L. J. , Ma, S. , Zhang, Y. , Nguyen, L. H. , Tickle, T. L. , Weingart, G. , Ren, B. , & Schwager, E. H. (2021). Multivariable association discovery in population‐scale meta‐omics studies. PLoS Computational Biology, 17, e1009442. 34784344 10.1371/journal.pcbi.1009442 PMC8714082

[ece310079-bib-0044] Marr, A. B. (1996). *Territoriality in Shackleford Island feral horses (*Equus caballus*)* . [Unpublished thesis]. Princeton University.

[ece310079-bib-0045] McKenzie, V. J. , Song, S. J. , Delsuc, F. , Prest, T. L. , Oliverio, A. M. , Korpita, T. M. , Alexiev, A. , Amato, K. R. , Metcalf, J. L. , Kowalewski, M. , Avenant, N. L. , Link, A. , Di Fiore, A. , Seguin‐Orlando, A. , Feh, C. , Orlando, L. , Mendelson, J. R. , Sanders, J. , & Knight, R. (2017). The effects of captivity on the mammalian gut microbiome. Integrative and Comparative Biology, 57, 690–704. 28985326 10.1093/icb/icx090 PMC5978021

[ece310079-bib-0046] McMurdie, P. J. , & Holmes, S. (2013). Phyloseq: An R package for reproducible interactive analysis and graphics of microbiome census data. PLoS One, 8, e61217. 23630581 10.1371/journal.pone.0061217 PMC3632530

[ece310079-bib-0047] McShea, W. J. , Monfort, S. L. , Hakim, S. , Kirkpatrick, J. , Liu, I. , Turner, J. W. , Chassy, L. , & Munson, L. (1997). The effect of immunocontraception on the behavior and reproduction of white‐tailed deer. Journal of Wildlife Management, 61, 560–569.

[ece310079-bib-0048] Mostl, E. , & Palme, R. (2002). Hormones as indicators of stress. Domestic Animal Endocrinology, 23, 67–74. 12142227 10.1016/s0739-7240(02)00146-7

[ece310079-bib-0049] National Climate Data Center (2022). http://www.ncdc.noaa.gov/oa/climate/climatedata.html#monthly

[ece310079-bib-0050] National Research Council . (2011). Guide for the care and use of laboratory animals (8th ed.). The National Academies Press. 21595115

[ece310079-bib-0051] Nearing, J. T. , Douglas, G. M. , Hayes, M. G. , MacDonald, J. , Desai, D. K. , Allward, N. , Jones, C. M. , Wright, R. J. , Dhanani, A. S. , & Comeau, A. M. (2022). Microbiome differential abundance methods produce different results across 38 datasets. Nature Communications, 13, 342. 10.1038/s41467-022-28034-z PMC8763921 35039521

[ece310079-bib-0052] Noguera, J. C. , Aira, M. , Pérez‐Losada, M. , Domínguez, J. , & Velando, A. (2018). Glucocorticoids modulate gastrointestinal microbiome in a wild bird. Royal Society Open Science, 5, 171743. 29765642 10.1098/rsos.171743 PMC5936907

[ece310079-bib-0053] Nuñez, C. M. V. (2000). Mother‐young relationships in feral horses (Equus caballus): Implications for the function of development in mammals. Princeton University. http://search.proquest.com/pqdtft/advanced?accountid=13314

[ece310079-bib-0054] Nuñez, C. M. V. , Adelman, J. S. , Carr, H. A. , Alvarez, C. M. , & Rubenstein, D. I. (2017). Lingering effects of contraception management on feral mare (*Equus caballus*) fertility and social behavior. Conservation Physiology, 5, cox018. 29977561 10.1093/conphys/cox018 PMC6007543

[ece310079-bib-0055] Nuñez, C. M. V. , Adelman, J. S. , Mason, C. , & Rubenstein, D. I. (2009). Immunocontraception decreases group fidelity in a feral horse population during the non‐breeding season. Applied Animal Behaviour Science, 117, 74–83.

[ece310079-bib-0056] Nuñez, C. M. V. , Adelman, J. S. , Smith, J. , Gesquiere, L. R. , & Rubenstein, D. I. (2014). Linking social environment and stress physiology in feral mares (*Equus caballus*): Group transfers elevate fecal cortisol levels. General and Comparative Endocrinology, 196, 26–33. 24275609 10.1016/j.ygcen.2013.11.012

[ece310079-bib-0057] Oksanen, J. , Blanchet, F. G. , Kindt, R. , Legendre, P. , Minchin, P. R. , O'Hara, R. B. , Simpson, G. L. , Solymos, P. , Stevens, M. H. H. , & Wagner, H. (2018). Vegan: Community ecology package . R package version 2.3‐5.

[ece310079-bib-0058] Ozato, N. , Saito, S. , Yamaguchi, T. , Katashima, M. , Tokuda, I. , Sawada, K. , Katsuragi, Y. , Kakuta, M. , Imoto, S. , & Ihara, K. (2019). *Blautia* genus associated with visceral fat accumulation in adults 20–76 years of age. NPJ Biofilms and Microbiomes, 5, 28. 31602309 10.1038/s41522-019-0101-x PMC6778088

[ece310079-bib-0059] Pollock, J. (1980). Behavioural ecology and body condition changes in New Forest ponies. Scientific Publications of the RSPCA, 6, 1–118.

[ece310079-bib-0060] Price, M. N. , Dehal, P. S. , & Arkin, A. P. (2010). FastTree 2 – Approximately maximum‐likelihood trees for large alignments. PLoS One, 5, e9490. 20224823 10.1371/journal.pone.0009490 PMC2835736

[ece310079-bib-0061] R Core Team . (2022). R: A language and environment for statistical computing. R Foundation for Statistical Computing.

[ece310079-bib-0062] Ransom, J. I. , Cade, B. S. , & Hobbs, N. T. (2010). Influences of immunocontraception on time budgets, social behavior, and body condition in feral horses. Applied Animal Behaviour Science, 124, 51–60.

[ece310079-bib-0063] Rubenstein, D. I. (1981). Behavioural ecology of island feral horses. Equine Veterinary Journal, 13, 27–34.

[ece310079-bib-0064] Rubenstein, D. I. (1986). Ecology and sociality in horses and zebras. In D. I. Rubenstein & R. W. Wrangham (Eds.), Ecological aspects of social evolution, birds and mammals (pp. 282–302). Princeton University Press.

[ece310079-bib-0065] Rubenstein, D. I. (1994). The ecology of female social behavior in horses, zebras, and asses. In P. J. Jarman & A. Rossiter (Eds.), Animal societies: Individuals, interactions, and organization (pp. 13–28). Physiology and Ecology Japan.

[ece310079-bib-0066] Rubenstein, D. I. , & Feinstein, L. H. (2021). Bothersome flies: How free‐ranging horses reduce harm while maintaining nutrition. Frontiers in Ecology and Evolution, 9, 659570.

[ece310079-bib-0067] Rubenstein, D. I. , & Nuñez, C. M. V. (2009). Sociality and reproductive skew in horses and zebras. In R. Hager & C. B. Jones (Eds.), Reproductive skew in vertebrates: Proximate and ultimate causes (pp. 196–226). Cambridge University Press.

[ece310079-bib-0068] Rutberg, A. T. (1990). Intergroup transfer in Assateague pony mares. Animal Behaviour, 40, 945–952.

[ece310079-bib-0069] Sacco, A. G. (1977). Antigenic cross‐reactivity between human and pig zona pellucida. Biology of Reproduction, 16, 164–173. 401654 10.1095/biolreprod16.2.164

[ece310079-bib-0070] Schoster, A. , Mosing, M. , Jalali, M. , Staempfli, H. R. , & Weese, J. S. (2016). Effects of transport, fasting and anaesthesia on the faecal microbiota of healthy adult horses. Equine Veterinary Journal, 48, 595–602. 26122549 10.1111/evj.12479

[ece310079-bib-0071] Stothart, M. R. , Bobbie, C. B. , Schulte‐Hostedde, A. I. , Boonstra, R. , Palme, R. , Mykytczuk, N. C. , & Newman, A. E. (2016). Stress and the microbiome: Linking glucocorticoids to bacterial community dynamics in wild red squirrels. Biology Letters, 12, 20150875. 26740566 10.1098/rsbl.2015.0875 PMC4785925

[ece310079-bib-0072] Stothart, M. R. , Greuel, R. J. , Gavriliuc, S. , Henry, A. , Wilson, A. J. , McLoughlin, P. D. , & Poissant, J. (2021). Bacterial dispersal and drift drive microbiome diversity patterns within a population of feral hindgut fermenters. Molecular Ecology, 30, 555–571. 33231332 10.1111/mec.15747

[ece310079-bib-0073] Tung, J. , Barreiro, L. B. , Burns, M. B. , Grenier, J.‐C. , Lynch, J. , Grieneisen, L. E. , Altmann, J. , Alberts, S. C. , Blekhman, R. , & Archie, E. A. (2015). Social networks predict gut microbiome composition in wild baboons. eLife, 4, e05224. 25774601 10.7554/eLife.05224 PMC4379495

[ece310079-bib-0074] Turnbaugh, P. J. , Ridaura, V. K. , Faith, J. J. , Rey, F. E. , Knight, R. , & Gordon, J. I. (2009). The effect of diet on the human gut microbiome: A metagenomic analysis in humanized gnotobiotic mice. Science Translational Medicine, 1, 6ra14. 10.1126/scitranslmed.3000322 PMC2894525 20368178

[ece310079-bib-0075] Turner, J. W., Jr. , Liu, I. K. M. , Flanagan, D. R. , Rutberg, A. T. , & Kirkpatrick, J. F. (2007). Immunocontraception in wild horses: One inoculation provides two years of infertility. The Journal of Wildlife Management, 71, 662–667.

[ece310079-bib-0076] Vaziri, G. J. , Jones, M. M. , Carr, H. A. , & Nuñez, C. M. V. (2022). *vaziri et al_metadata_20201231*. Open Science Framework. https://osf.io/rhz7a/

[ece310079-bib-0077] Ward, A. , & Webster, M. (2016). Sociality: The behaviour of group‐living animals. Springer Cham.

[ece310079-bib-0078] Wasser, S. K. , Hunt, K. E. , Brown, J. L. , Cooper, K. , Crockett, C. M. , Bechert, U. , Millspaugh, J. J. , Larson, S. , & Monfort, S. L. (2000). A generalized fecal glucocorticoid assay for use in a diverse array of nondomestic mammalian and avian species. General and Comparative Endocrinology, 120, 260–275. 11121291 10.1006/gcen.2000.7557

[ece310079-bib-0079] Wickham, H. (2016). ggplot2: Elegant graphics for data analysis. Springer.

[ece310079-bib-0080] Willis, P. (1994). Equine immunoconcentraception using porcine zona‐pellucida – A new method for remote delivery and characterization of the immune‐response. Journal of Equine Veterinary Science, 14, 429.

[ece310079-bib-0081] Wu, G. D. , Chen, J. , Hoffmann, C. , Bittinger, K. , Chen, Y.‐Y. , Keilbaugh, S. A. , Bewtra, M. , Knights, D. , Walters, W. A. , & Knight, R. (2011). Linking long‐term dietary patterns with gut microbial enterotypes. Science, 334, 105–108. 21885731 10.1126/science.1208344 PMC3368382

[ece310079-bib-0082] Yildirim, S. , Yeoman, C. J. , Sipos, M. , Torralba, M. , Wilson, B. A. , Goldberg, T. L. , Stumpf, R. M. , Leigh, S. R. , White, B. A. , & Nelson, K. E. (2010). Characterization of the fecal microbiome from non‐human wild primates reveals species specific microbial communities. PLoS One, 5, e13963. 21103066 10.1371/journal.pone.0013963 PMC2980488

[ece310079-bib-0083] Zaiss, M. M. , & Harris, N. L. (2016). Interactions between the intestinal microbiome and helminth parasites. Parasite Immunology, 38, 5–11. 26345715 10.1111/pim.12274 PMC5019230

[ece310079-bib-0084] Zaneveld, J. R. , Burkepile, D. E. , Shantz, A. A. , Pritchard, C. E. , McMinds, R. , Payet, J. P. , Welsh, R. , Correa, A. M. , Lemoine, N. P. , & Rosales, S. (2016). Overfishing and nutrient pollution interact with temperature to disrupt coral reefs down to microbial scales. Nature Communications, 7, 1–12. 10.1038/ncomms11833 PMC4899628 27270557

[ece310079-bib-0085] Zaneveld, J. R. , McMinds, R. , & Thurber, R. V. (2017). Stress and stability: Applying the Anna Karenina principle to animal microbiomes. Nature Microbiology, 2, 1–8. 10.1038/nmicrobiol.2017.121 28836573

