# Supplementary material for: Out of the stable: Social disruption and concurrent shifts in the feral mare (Equus caballus) fecal microbiota
Source: Ecol Evol. 2023 May 11;13(5):e10079. doi: 10.1002/ece3.10079 (PMC10175550; doi:10.1002/ece3.10079)
Supplement: Supplementary file 3 — Table S1 [file ECE3-13-e10079-s006.docx]

**Supporting Information Table 1.** Mare sampling regimes and immunocontraception treatment during study. All mares were previously treated at some point between 2000 and 2009.

| **Mare** | **Year(s) observed** | **Initial band stallion** | **Island region** | **PZP treatment during study** |
| --- | --- | --- | --- | --- |
| Alexa | 2016 | JFK | West | Treated |
| Ariel | 2015 | Shakespeare | West | Untreated |
| Biff | 2015 & 2016 | Abu/Abu and Atari | West | Untreated |
| Bridget | 2015 & 2016 | Dilbert/JFK | West | Untreated |
| Darcy | 2016 | Noel | West | Untreated |
| Delphi | 2016 | Shakespeare | West | Untreated |
| Djibouti | 2015 | Homer and Dale | West | Untreated |
| Donoma | 2016 | Katsu | Central | Untreated |
| Dusty | 2015 & 2016 | Sebastian | Central | Untreated |
| Hardee | 2016 | Katsu | Central | Untreated |
| Hezakiah | 2016 | Katsu | Central | Untreated |
| Hoorah | 2015 & 2016 | Noah/Sebastian | Central | Untreated |
| Julie | 2016 | Abu and Atari | West | Untreated |
| Juniper | 2016 | Judd | West | Untreated |
| Kelty | 2016 | Simon | Central | Untreated |
| Larissa | 2015 & 2016 | Dilbert | East | Untreated |
| Lassie | 2016 | Largo | West | Untreated |

| **Mare** | **Year(s) observed** | **Initial band stallion** | **Island region** | **PZP treatment during study** |
| --- | --- | --- | --- | --- |
| Liani | 2016 | JFK | West | Untreated |
| Noir | 2016 | Cataan and Noah | Central | Untreated |
| Paula | 2015 & 2016 | Katsu | East | Untreated |
| Sadie | 2015 & 2016 | Homer and Dale | Central | Treated  (2015 & 2016) |
| Sarah | 2015 & 2016 | Dilbert | West | Untreated |
| Sawathu | 2016 | Shakespeare | West | Untreated |
| Serenac | 2015 & 2016 | Zim/Waco | Central | Untreated |
| Shira | 2016 | JFK | West | Untreated |
| Tatya | 2016 | Waco | Central | Untreated |
| Texas | 2015 & 2016 | Dilbert/Katsu | East/Central | Untreated |
| Tiger | 2015 | Kabuki/Dilbert | West | Untreated |
| Wallace | 2015 & 2016 | Simon | Central | Untreated |
| Wanaka | 2016 | Shakespeare | West | Untreated |
