# Supplementary material for: Out of the stable: Social disruption and concurrent shifts in the feral mare (Equus caballus) fecal microbiota
Source: Ecol Evol. 2023 May 11;13(5):e10079. doi: 10.1002/ece3.10079 (PMC10175550; doi:10.1002/ece3.10079)
Supplement: Supplementary file 4 — Table S2 [file ECE3-13-e10079-s001.docx]

**Supporting Information Table 2.** PZP treatment and sampling information for mares (*Equus caballus*) that changed groups on Shackleford Banks, NC (2015-2016).

| **Mare** | **Most recent PZP treatment** | **Estimated (median) date of group change** | **Fecal collection date** | **Estimated no. days between group change and fecal collection** |
| --- | --- | --- | --- | --- |
| Hoorah | 2009 | 06/13/2015 | 06/30/2015 | 16.5 |
| Larissa | 2006 | 06/12/2015 | 07/15/2015 | 33 |
| Serenac | 2009 | 07/09/2015 | 07/17/2015 | 8 |
| Texas | 2006 | 06/12/2015 | 06/27/2015 | 15 |
| Texas | 2006 | 06/12/2015 | 07/15/2015 | 33 |
| Tiger | 2006 | 06/11/2015 | 07/16/2015 | 34.5 |
| Tiger | 2006 | 06/11/2015 | 07/21/2015 | 39.5 |
| Darcy | 2005 | 06/06/2016 | 06/27/2016 | 20.5 |
| Darcy | 2005 | 06/06/2016 | 07/25/2016 | 48.5 |
| Hoorah | 2009 | 07/16/2016 | 07/21/2016 | 4.5 |
| Kelty | 2009 | 06/28/2016 | 07/15/2016 | 16.5 |
| Larissa | 2006 | 06/01/2016 | 06/17/2016 | 15.5 |
| Lassie | 2008 | 06/01/2016 | 06/16/2016 | 15 |
| Lassie | 2008 | 06/19/2016 | 06/23/2016 | 3.5 |
| Sawathu | 2009 | 06/27/2016 | 07/01/2016 | 3.5 |
| Tatya | 2006 | 06/28/2016 | 07/01/2016 | 1.5 |
