# Supplementary material for: Out of the stable: Social disruption and concurrent shifts in the feral mare (Equus caballus) fecal microbiota
Source: Ecol Evol. 2023 May 11;13(5):e10079. doi: 10.1002/ece3.10079 (PMC10175550; doi:10.1002/ece3.10079)
Supplement: Supplementary file 5 — Table S3 [file ECE3-13-e10079-s004.docx]

**Supporting Information Table 3.** Differentially abundant bacterial families detected by both ANCOM-BC and MaAsLin2 in mare fecal samples from different years and regions, organized from most differentially absent (detected less than expected by chance) to differentially abundant (detected more than expected by chance). Families are identified by the most specific taxonomic level for which a classification could be obtained.

| Comparison | FamilyID | Mares with family present | Beta | SE | FDR-corrected P | LFC | SE | FDR-corrected P |
| --- | --- | --- | --- | --- | --- | --- | --- | --- |
| 2016 vs. 2015 | Erysipelotrichaceae | 35 | -2.02 | 0.36 | 0.00 | -1.18 | 0.21 | 0.00 |
|  | Mycoplasmataceae | 52 | -1.50 | 0.21 | 0.00 | -0.84 | 0.13 | 0.00 |
|  | Myxococcaceae | 23 | -1.44 | 0.41 | 0.02 | -0.71 | 0.28 | 0.04 |
|  | Butyricicoccaceae | 12 | -1.44 | 0.24 | 0.00 | -0.56 | 0.21 | 0.03 |
|  | Christensenellaceae | 52 | -1.06 | 0.28 | 0.01 | -0.51 | 0.17 | 0.02 |
|  | Anaerovoracaceae | 52 | -1.03 | 0.21 | 0.00 | -0.55 | 0.12 | 0.00 |
|  | Order: Bacteroidales, Family F082 | 52 | -0.99 | 0.25 | 0.01 | -0.55 | 0.14 | 0.00 |
|  | Erysipelatoclostridiaceae | 52 | 0.49 | 0.13 | 0.01 | 0.52 | 0.11 | 0.00 |
|  | Acholeplasmataceae | 52 | 1.42 | 0.24 | 0.00 | 1.12 | 0.20 | 0.00 |
| Central vs. Eastern Region | Phylum: Firmicutes, uncultured family | 14 | -1.34 | 0.32 | 0.00 | -1.13 | 0.30 | 0.01 |
| Western vs. Eastern Region | Clostridiaceae | 43 | -2.87 | 0.92 | 0.05 | -1.81 | 0.53 | 0.02 |
|  | Phylum: Firmicutes, uncultured family | 14 | -1.60 | 0.33 | 0.00 | -1.17 | 0.31 | 0.01 |
