# Supplementary material for: Out of the stable: Social disruption and concurrent shifts in the feral mare (Equus caballus) fecal microbiota
Source: Ecol Evol. 2023 May 11;13(5):e10079. doi: 10.1002/ece3.10079 (PMC10175550; doi:10.1002/ece3.10079)
Supplement: Supplementary file 6 — Table S4 [file ECE3-13-e10079-s005.docx]

**Supporting Information Table 4.** Differentially abundant bacterial genera detected by both ANCOM-BC and MaAsLin2 in mare fecal samples from different years and regions, organized from most differentially absent (detected less than expected by chance) to differentially abundant (detected more than expected by chance). Genera are identified by the most specific taxonomic level for which a classification could be obtained.

|  |  |  | MaAsLin2 Stats | | | ANCOM-BC Stats | | |
| --- | --- | --- | --- | --- | --- | --- | --- | --- |
| Comparison | Genus ID | N Mares with genus present | Beta | SE | FDR-corrected *P* | LFC | SE | FDR-corrected *P* |
| 2016 vs. 2015 | Family: Erysipelotrichaceae, *uncultured genus* | 26 | -2.37 | 0.26 | 0.00 | -1.27 | 0.20 | 0.00 |
|  | Family: Anaerovoracaceae, genus in *Family XIII_AD3011 group* | 43 | -1.93 | 0.36 | 0.00 | -1.08 | 0.19 | 0.00 |
|  | *Oribacterium* | 15 | -1.80 | 0.20 | 0.00 | -0.99 | 0.21 | 0.00 |
|  | *Anaerovorax* | 52 | -1.57 | 0.35 | 0.00 | -0.71 | 0.17 | 0.00 |
|  | *Mycoplasma* | 52 | -1.50 | 0.21 | 0.00 | -0.75 | 0.12 | 0.00 |
|  | Order: Bacteroidales, Family F082, genus *F082* | 52 | -0.99 | 0.25 | 0.01 | -0.46 | 0.14 | 0.01 |
|  | *Anaeroplasma* | 52 | 1.34 | 0.29 | 0.00 | 1.12 | 0.17 | 0.00 |
| Central vs. Eastern Region | Family: Erysipelotrichaceae, *uncultured genus* | 26 | 1.69 | 0.41 | 0.01 | 0.94 | 0.27 | 0.03 |
| Western vs. Eastern Region | Phylum: Firmicutes, *uncultured genus* | 14 | -1.60 | 0.33 | 0.00 | -1.02 | 0.16 | 0.02 |
